# MEsh FIxation in Laparoendsocopic Repair of Large M3 inguinal hernias: multicenter, double-blinded, randomized controlled trial—study protocol for a MEFI Trial

**DOI:** 10.1186/s13063-023-07601-9

**Published:** 2023-09-05

**Authors:** Mateusz Zamkowski, Maciej Śmietański

**Affiliations:** 1 General Surgery and Hernia Centre, Swissmed Hospital, Gdańsk, Poland; 2 2nd Department of Radiology, Medical University of Gdańsk, Gdańsk, Poland

**Keywords:** Hernia, TAPP, TEP, Laparoendoscopy, Miniinvasive, Mesh, Medial hernia, Groin hernia, Inguinal hernia

## Abstract

**Background:**

International guidelines of groin hernia treatment strongly recommend to fixate the mesh in large M3 medial defects during TAPP/TEP procedures. The main purpose of fixation is to decrease the recurrence rate which is alarmingly high in case of those defects. In 2022, a team consisting of hernia surgeons and scientists from universities of technology conducted an experimental study with the use of 3D groin model to verify the hypothesis that fixation is not necessary in above cases. Experiment showed that rigid and anatomically shaped meshes are able to maintain its position in the groin without fixation. Similar conclusions were recently published in Swedish database registry analysis. To confirm above results, we decided to conduct a multicenter randomized controlled trial.

**Methods:**

Main objective of MEFI Trial is to verify the hypothesis that non-fixation of spatial, standard polypropylene meshes *is non-inferior* to fixation of flat, polypropylene lightweight meshes in M3 hernias by laparoendoscopic approach. Eleven large surgery centers in Poland having proficiency in laparoendoscopic groin hernia repairs were recruited for this study. Recurrence in 12-month follow-up was set as a primary endpoint. Pain sensation (Visual Analog Scale) and incidence of other complications (hematoma, seroma, SSI) were also noted. Based on the statistical analysis, minimal sample size in both arms was established at 83–102. The first arm (control) consists of patients undergoing a repair with the use of a flat, macroporous mesh with fixation using histoacryl glue. In the second arm, patients will be operated with the use of anatomically shaped, standard-weight mesh without fixation. Study will be double-blinded (patient/surgeon). After the dissection of preperitoneal space, surgeon will open a sealed envelope and find out which technique he will have to perform. Follow-up will be performed by Study Secretary (also blinded to the method used) via phone call 3 and 12 months after surgery.

**Discussion:**

Based on experimental study and recent registry analysis, we believe that the recurrence rate in both groups would be on the same level, giving hernia societies a strong argument for amending the guidelines.

**Trial registration:**

ClinicalTrials.gov NCT05678465. Registered on 10 January 2023.

## Administrative information

Note: the numbers in curly brackets in this protocol refer to SPIRIT checklist item numbers. The order of the items has been modified to group similar items (see http://www.equator-network.org/reporting-guidelines/spirit-2013-statement-defining-standard-protocol-items-for-clinical-trials/).

**Table:** 

Title {1}	MEsh FIxation in Laparoendsocopic Repair of Large M3 Inguinal Hernias—Multicenter, Double-blinded, Randomized Controlled Trial Acronym: MEFI TRIAL
Trial registration {2a and 2b}.	**Registry name:** MEsh FIxation in Laparoendoscopic Repair of Large M3 Inguinal Hernias (MEFI) **ClinicalTrials.gov Identifier:** NCT05678465
Protocol version {3}	19.01.2023 – version 2.0
Funding {4}	No funding is present. Authors and collaborators do not report any conflict of interests.
Author details {5a}	Zamkowski Mateusz, MD, PhD^1^ Śmietański Maciej MD, PhD, Prof^1,2^ ^1^General Surgery and Hernia Center, Swissmed Hospital in Gdańsk, Poland ^2^2nd Department of Radiology, Medical University of Gdańsk, Poland
Name and contact information for the trial sponsor {5b}	Sponsor-investigator: **Swissmed Hospital in Gdańsk** General Surgery and Hernia Centre Wileńska 44, 80–215 Gdańsk, Poland Mateusz Zamkowski, MD, PhD, email: zamek@wp.eu
Role of sponsor {5c}	The funding body had no role in the design of the protocol and will not have any role in the conduct of the study including collection, analysis, and interpretation of data, or the writing of the manuscript or the decision to publish.

## Introduction

### Background and rationale {6a}

Every year, more than 20 million people worldwide are operated on for inguinal hernias. The methods of choice are laparoendoscopic techniques if the appropriate equipment and trained personnel are available [1]. TAPP and TEP are gradually replacing classical methods (including the Lichtenstein method) as first-line treatment. Although in laparoendoscopic methods, mesh fixation is not recommended, with the exception of large direct hernias (M3). The 2018 international guidelines contain a strong recommendation for mesh fixation for M3 defects [1].

The basis for formulating this recommendation is the analysis of the German Hernia Registry (HerniaMed) published by Meyer et al. in 2017 on 11,230 cases [2]. During the multivariate analysis, it was noted that the recurrence rate in the case of large M3 inguinal hernias was alarmingly high, and the implant fixation significantly reduced the recurrence rate in those cases. Despite weak scientific evidence, the recommendation was upgraded to strong status by a panel of experts.

In 2022, a multidisciplinary team consisting of surgeons and scientists from the Warsaw and Cracow Universities of Technology conducted a research experiment aimed at checking the behavior of implants in the operating field under the highest possible intraabdominal pressure. Based on the previously constructed groin model, tests of implants used during laparoendoscopic repairs of inguinal hernias were carried out. Results confirm the hypothesis that in the case of large M3 hernias, the key role in maintaining the implant in the operating field is the type of mesh (stiffness, appropriate margin, spatial shape) rather than its fixation. Only stiffer, anatomically matched implants were able to maintain mechanical stability under pressure surges. Flat, macropore implants in the vast majority of the Herniamed registry were “shot” through the hernial orifices and lost their initial position, thus giving room for rapid recurrence [3].

In the same year, Novik et al. published data from the Swedish Hernia Registry, where, similarly to the work of Meyer et al., they analyzed the results of patients treated for inguinal hernias in 2005–2017 [4]. 25,190 patients were analyzed and a multifactorial analysis was used to correlate the type of implant used, the size of the hernia, and the risk of recurrence. Conclusions coincide with those presented by Zamkowski et al.

In order to confirm experimental results and conclusions contained in the analysis of Swedish hernia registries, a decision was made to conduct a multicenter, randomized, double-blind clinical trial. The aim will be to prove that the lack of fixation of a standard, anatomic spatial mesh is noninferior to the fixation of a light, macroporous flat mesh in the case of large M3 hernias operated laparoendoscopically.

Confirmation of the conclusions from experimental work and retrospective analysis of the Swedish hernia registry through a randomized controlled trial will give rise to a change or update of international guidelines for the treatment of inguinal hernias.

### Objectives {7}

Main objective of MEFI Trial is to verify the hypothesis that non-fixation of spatial, standard polypropylene meshes *is non-inferior* to fixation of flat, polypropylene lightweight meshes in M3 hernias by laparoendoscopic approach in terms of recurrence.

### Trial design {8}

**Fig. 1:**
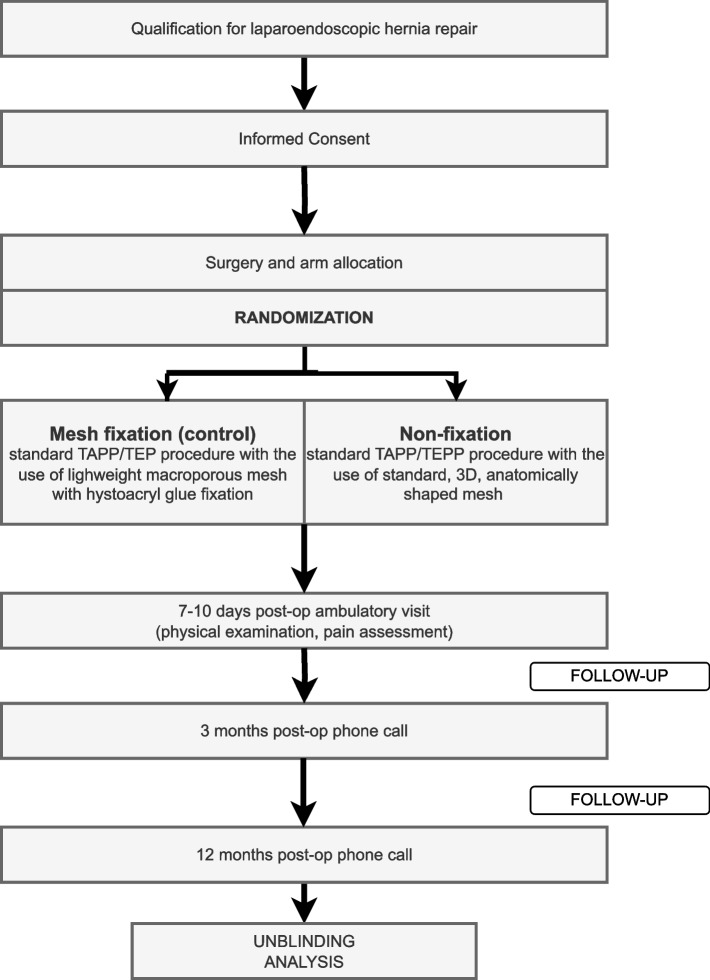
Flow-diagram of MEFI Trial

MEFI Trial is a prospective, non-inferiority, parallel, two-armed, multicenter, double-blinded randomized controlled trial to study outcomes of non-fixation of the mesh in terms of recurrence in large M3 defects during laparoendoscopic hernia repair techniques. Eleven large surgery centers in Poland, proficient in laparoendoscopic groin hernia repairs (performing at least 80 TAPP/TEP procedures a year), were selected for this study (Table 1 and Fig. 1). List of study sites (collaborators) is given in the Organizational Structure and Responsibilities section at the end of document. Recurrence rate in 12-month follow-up was set as a primary outcome.

**Table 1:** Schedule of enrolment, interventions, and assessments for the MEFI trial

	**Study period**					
	**Enrolment**	**Allocation**	**Post-allocation**			**Close-out (unblinding)**
**Timepoint**	*** − (1–2) weeks***	**0**	***7–10 days***	***3 months***	***12 months***	***12 months***
**Enrolment:**						
**Eligibility screen**	X					
**Informed consent**	X					
***Surgery (TAPP/TEP)***		X				
**Allocation**		X				
**Interventions:**						
***FIXATION of flat macroporous lightweight mesh—CONTROL***		X				
***NON-FIXATION of 3D, anatomical StdPPM***^a^		X				
**Assessments:**						
***Recurrence rate—primary endpoint***			X	X	X	X
***Pain sensation—secondary outcomes***			X	X	X	X
***Other complications***^b^***—other outcomes***			X	X	X	X

^a^Standard pure polypropylene mesh

^b^Other complications: hematoma, seroma, and surgical site infection

## Methods: participants, interventions, and outcomes

### Study setting {9}

Eleven large surgery centers in Poland, proficient in laparoendoscopic groin hernia repairs (performing at least 80 TAPP/TEP procedures a year), were selected for this study (Table 1 and Fig. 1). List of study sites (collaborators) is given in the Organizational Structure and Responsibilities section at the end of document. Recurrence rate in 12-month follow-up was set as a primary outcome. Swissmed Hospital in Gdańsk, Poland, is responsible for the coordination of MEFI Trial, as well as, for conducting the phone call follow-up protocol. Also, it is the central point of contact for study participants and surgeons. Study participants will be recruited in collaborating hospitals and in coordinating unit. Study Secretary and Study Chief will be known to participants and they will be given their e-mail and phone number to stay in constant contact. Study Chief and Study Director along with Study Secretary and two volunteers from collaborators centers will be form the Trial Steering Committee. The Trial Steering Committee will meet once a month to exchange information and evaluate recent data. Study group will also have access to WhatsApp group and Facebook closed group to exchange comments/observations and provide organizational support. Study Chief, Study Director, and Study Secretary will be available to all study participants and other collaborators 24/7. Data will be managed by each surgeon designated from collaborators centers. All designated surgeons will form the Data Management Team.

### Eligibility criteria {10}

Patients undergoing elective groin hernia repair will be initially recruited for the study. Inclusion and exclusion criteria are listed in Table 2. As M3 Hernias are difficult to diagnose before the surgery, all patients scheduled for laparoendoscopic repairs will be asked to participate. Final recruitment to the study will take place after placing visual track and measuring the size of defect. Informed verbal and written consent will be obtained by the surgeon performing the operation. GDPR (General Data Protection Regulation) consent will be taken by the medical secretary on admission to the hospital.

**Table 2:** Inclusion and exclusion criteria in the MEFI trial

Inclusion criteria	Exclusion criteria
Elective groin hernia repair	Age < 18 years
Age > 18 years	Eemergency surgery (incarcerated hernia)
Male and female patients can participate	Contaminated surgical field
M3 or M3 + L1-3 (EHS classification) groin hernia confirmed during surgery	Recurrent hernia
Eligibility for laparoendoscopic repair	Extremely large scrotal hernias with the need of other abdominal compartment syndrome (ACS) preventive procedures (botulin injection, bowel resection, preoperative progressive pneumoperitoneum (PPP))
Signed written informed consent	

### Who will take informed consent? {26a}

Informed verbal and written consent will be obtained by the surgeon performing the operation.

### Additional consent provisions for collection and use of participant data and biological specimens {26b}

On the consent form, participants will be asked if they agree to use their data should they choose to withdraw from the trial. Participants will also be asked for permission for the research team to share relevant data with people from the Universities taking part in the research or from regulatory authorities, where relevant. This trial does not involve collecting biological specimens for storage.

## Interventions

### Explanation for the choice of comparators {6b}

First arm, also serving as control group, will consist of patients operated with the use of flat, macroporous, lightweight mesh fixated with the use of histoacryl glue. This method is currently recommended for large M3 hernias in 2018 European Hernia Society Guidelines. Second arm (experimental group) will consist of patients operated with the use of anatomically shaped, large, standard polypropylene mesh (Dextile Anatomical Mesh – MEDTRONIC™ (Dublin, Ireland) or 3D Max Mesh – BD™ (New Jersey, USA)) without the use of fixation material. The effectiveness of method is supported by recent publications published in 2022 by Zamkowski et al. and Novik et al. [2, 3].

### Intervention description {11a}

**Fig. 2:**
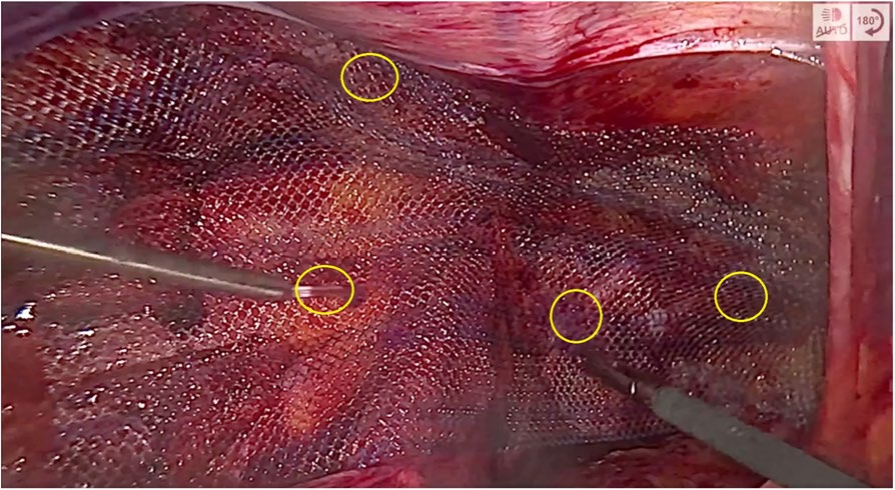
Landmarks to place histoacryl glue during mesh fixation

As M3 hernias are difficult to diagnose before the surgery, all patients scheduled for laparoendoscopic repairs will be asked to participate. After the dissection, reduction of hernia sack, and creation of preperitoneal space, the surgeon will open a sealed envelope and find out what technique he/she will have to perform and participants will be randomly allocated to one arm of the study. Dissection of preperitoneal space and placing the mesh in the groin area are considered to be the most important stage of the procedure—every surgeon will have to take visual record of that part of surgery to make sure proper technique was applied. Patients will not be aware of the method used during surgery. When using fixation material with histoacryl glue, it will be placed in previously predefined places (Fig. 2). Before recruiting patients, a 2-day “bootcamp” meeting was scheduled. Each center designated one surgeon responsible for conducting the trial in their hospital and sent him/her to attend the meeting. The meeting consisted of lectures on the first day and exhibition surgeries performed by experts on the second day. A meeting took place to unify the surgery technique and make sure every center will follow the same protocol. After operation, surgeon will fill the study form and put it in a sealed envelope. All patients in both arms will be given standard recommendation to avoid intensive physical activity for 2 weeks post-op. Standard wound care will be explained to patients in both groups.

### Criteria for discontinuing or modifying allocated interventions {11b}

Participants of the study can withdraw their consent to take part at any time. The principal investigator may exclude patients from the study, if patients’ safety is at risk. In order to generate a meaningful database, excluded patients can be replaced by the recruitment of new patients.

### Strategies to improve adherence to interventions {11c}

Adherence to the follow-up visit schedule is promoted by facilitating the study visit within 7–10 days after surgery and telephone interview at 3 months and 12 months post-op.

### Relevant concomitant care permitted or prohibited during the trial {11d}

All patients in both arms will be given standard recommendation to avoid intensive physical activity for 2 weeks post-op. Standard wound care will be explained to patients in both groups.

### Provisions for post-trial care {30}

There are no provisions for post-trial care.

### Outcomes {12}

Participants will complete measurements for primary and secondary endpoints at baseline, 7 days post-baseline (post-intervention), 3 months, and 1 year post-baseline (follow-up) (Table 1).

#### Primary outcome

The primary endpoint is the recurrence rate in 1-year follow-up.

#### Secondary outcomes

Besides primary outcome secondary outcomes will also be noted.

1. Pain sensation assessed with Visual Analog Scale measured during hospital stay, 7 days post-op, 3 months, and 12 months after surgery.

2. Incidence of other complications (seroma, hematoma, infections) assessed during hospital stay, 7 days post-op, 3 months, and 12-months after surgery.

7 day post-op visit will be performed by a surgeon blinded to the arm allocation. During the visit, another form will be filled and placed in encoded envelope. Three months post-op and 1-year post-op follow-up, a phone call will be performed by the Study Secretary blinded to the allocation. Telephone call after inguinal hernia surgery is a verified and validated form of follow-up [5, 6]. If there are any concerns, the patient will be asked to attend an outpatient appointment.

Participant demographics including general medical history, body mass index, co-morbidities, age, sex, smoking status, history of hernia repairs, and family history of hernias will be noted and placed in sealed coded envelopes.

### Participant timeline {13}

All patients undergoing elective inguinal hernia repair, meeting the inclusion criteria for the study, will be asked to participate and given informed written consent. After creating a pneumoperitoneum and placing a vision trocar, the hernia defect will be measured. If the presence of a simple M3 hernia (or a complex M3 hernia with a component of an oblique hernia) is confirmed, full inclusion in the study will take place. After the next stages of the operation, i.e., placement of working trocars, release of adhesions, and dissection of the preperitoneal space with reduction of the hernial sac, the stage of random allocation to the study arms will take place by opening the envelope and learning the technique to be used in a given participant. After the procedure, the surgeon will fill out the researcher’s card and place the operating protocol in a prepared coded envelope. The participant will receive information about full inclusion in the study and full follow-up. A follow-up visit 7–10 days after the surgery will take place in the hospital outpatient clinic, during which further information about the health condition will be collected, and a physical examination will be performed. Both groups will receive the same standard postoperative recommendations. Subsequent follow-ups (3 months and 12 months after) will take place through a telephone conversation made by study secretary blinded to the method used. Detailed timeline is presented in Table 1.

### Sample size {14}

Statistical analysis was performed to adjust the sample sizes. The trial was planned as non-inferiority study with test power = 80%,* p*-value = 0.05, and threshold for clinical significance = 8%. A percentage loss of 10% was assumed. Recurrence rate was assumed to be 4% based on literature data. Based on those assumptions, minimal sample size in both arms was established at 83–102.

### Recruitment {15}

Recruitment commenced in February 2023. Participants are recruited in centers managed by the Sponsor and by Collaborators. Despite inguinal hernias being a common disease and over 60–70 thousand inguinal hernia repairs are being performed in Poland annually, M3 and complex hernias are rather rare. To obtain required number of participants, a multicenter study is needed and that is why 11 large surgery centers in Poland, proficient in laparoendoscopic groin hernia repairs (performing at least 80 TAPP/TEP procedures a year), were selected for this study. Patients will be encouraged to complete follow-up via talk and discussion with surgeon. 10% of lost to follow-up is predicted.

## Assignment of interventions: allocation

### Sequence generation {16a}

Randomization will be performed with the use of the Clinical Trial Randomization Tool (National Cancer Institute) on 1:1 basis by Study Secretary. Based on randomization, 204 envelopes will be prepared, each with a unique code number. Inside, apart from blank research cards to be completed, there will also be a smaller envelope with the type of technique to be used during the procedure. Participants, the Study Secretary, and surgeons will be blinded in terms of allocation to individual arms (until the envelope is opened). Unblinding will take place at the end of the trial via phone call (or ambulatory visit if one will be scheduled) by Study Secretary.

### Concealment mechanism {16b}

During the surgery, when the defect size is measured, the patient will be fully included in the study if M3 hernia is present. After the dissection of preperitoneal space (and establishing crucial checkpoints), surgeon will open a sealed, random envelope and find out what technique he/she will have to perform.

### Implementation {16c}

Enrolment will be performed by Study Secretary. Each prepared envelope will have specific number previously generated by a computer program and will be associated with the technique performed (fixation/non-fixation). The name of the technique and, therefore, assignment to intervention will be placed in the proper envelope. Surgeon will not be aware of the technique he should perform before opening the envelope.

## Assignment of interventions: blinding

### Who will be blinded {17a}

Trial participants and Study Secretary performing phone call follow-up visit will be blinded to the method used. Surgeon will be blinded for as long as possible (after the dissection, reduction of hernia sack, and creation of preperitoneal space are achieved).

### Procedure for unblinding if needed {17b}

Unblinding will take place at the end of the trial (12 months) during ambulatory visit or phone call.

## Data collection and management

### Plans for assessment and collection of outcomes {18a}

Each center designated one surgeon responsible for conducting the trial in their hospital and sent him/her to attend the meeting. Data will be collected with the use of questionnaires exclusively by members of the research team (surgeons and the Study Secretary).

### Plans to promote participant retention and complete follow-up {18b}

Patients will be encouraged to complete follow-up via talk and discussion with the surgeon. Research Team will monitor the participants to ensure follow-up completion, including email and SMS reminders. 10% of lost to follow-up is predicted.

### Data management {19}

Only study investigators will have access to trial information and dataset. Personal information about enrolled participants will be collected in closed envelope in the hospital registry. Data will be held for 20 years after the trial. To protect confidentiality, no name/surname will be used. Each participant will be assigned a code number impossible to decode without a proper list. Participants will not be identified in the resulting manuscripts or reports. The Trial Steering Committee will have access to interim results and make the final decision to terminate the trial. Interim analysis will take place in half-way of the trial.

### Confidentiality {27}

Personal information about enrolled participants will be collected in closed envelope in hospital registry. Data will be held for 20 years after the trial. To protect confidentiality, no name/surname will be used. Each participant will have a code number impossible to decode without proper list. Participants will not be identified in the resulting manuscripts or reports.

### Plans for collection, laboratory evaluation, and storage of biological specimens for genetic or molecular analysis in this trial/future use {33}

No biological specimens will be stored.

## Statistical methods

### Statistical methods for primary and secondary outcomes {20a}

All statistical calculations will be performed using the statistical suite StatSoft Inc. (2014) STATISTICA (data analysis software system) version 12.0 (www.statsoft.com) and an Excel spreadsheet. Continuous variables will be characterized by arithmetic mean, standard deviation, median, minimum and maximum (range), and 95% CI (confidence interval). Qualitative variables, on the other hand, will be presented using the frequency and percentage values (percentage). The Shapiro–Wilk W test will be used to check whether the quantitative variable came from a normally distributed population. The Leven (Brown-Forsythe) test will be used to test the hypothesis of equal variances. Significance of differences between the two groups (model of unrelated variables) will be tested by the tests of significance of differences: Student’s *t*-test (or, in the absence of homogeneity of variance, the Welch test) or the Mann–Whitney U test (if the conditions of applicability of the Student’s *t*-test are not met or for variables measured on ordinal scale). Significance of differences between more than two groups will be checked by *F* test (ANOVA) or Kruskal–Wallis test (if the conditions of ANOVA applicability are not met). If statistically significant differences between the groups are obtained, post hoc tests will be used (Tukey’s test for *F*, Dunn’s test for Kruskal–Wallis). Chi-square tests of independence will be used for categorical variables (using Yates correction for cell counts below 10, checking Cochran conditions, Fisher’s exact test, respectively). In order to determine the relationship, strength, and direction between the variables, correlation analysis will be applied by calculating the Pearson and/or Spearman correlation coefficients. *P* = 0.05 will be assumed as the significance level in all calculations.

The trial was planned as non-inferiority study with test power = 80%, *p*-value = 0.05, and threshold for clinical significance = 8%. A percentage loss of 10% was assumed. Recurrence rate was assumed to be 4% based on literature data. Based on those assumptions, minimal sample size in both arms was established at 83–102. To ensure sufficient participant numbers at completion of the trial, sample size was established at 204 participants. To maintain the integrity and clarity of our results, we chose to focus on our primary analysis. Given the focused nature of our primary research question and to maintain the power of our statistical analyses, we opted not to divide our sample further into subgroups.

### Interim analyses {21b}

Trial Steering Committee will have access to interim results and make the final decision to terminate the trial. Interim analysis will take place in half-way of the trial.

### Methods for additional analyses (e.g., subgroup analyses) {20b}

No additional analyses will be made.

### Methods in analysis to handle protocol non-adherence and any statistical methods to handle missing data {20c}

Most important stages of the surgical procedures will be recorded to make sure proper technique was applied. Video files will be sent to a dedicated protected server and files will be named after participant code number. Trial Steering Committee will have access to those files and will evaluate them once a month to make sure no crucial errors in technique are made.

### Plans to give access to the full protocol, participant-level data, and statistical code {31c}

The datasets analyzed during the current study and statistical code are available from the corresponding author on reasonable request, as is the full protocol.

## Oversight and monitoring

### Composition of the coordinating center and trial steering committee {5d}

Study Secretary and Study Chief will be known to participants and will be given their e-mail and phone number to stay in constant contact. Swissmed Hospital is the coordinating center as a founder of the trial and serves as the main hub for overseeing the trial operations. Its roles and responsibilities:

1. Oversee the day-to-day operations of the trial.

2. Coordinate with participating centers for patient recruitment and data collection.

3. Ensure that the trial adheres to the protocol and regulatory requirements.

4. Address any logistical challenges and provide necessary resources.

Study Chief and Director, along with Study Secretary and two volunteers collaborators, will be forming Trial Steering Committee. Roles and responsibilities of the Trial Steering Committee:

1. Review and guide the overall direction of the trial.

2. Monitor trial progress and ensure objectives are being met.

3. Address any major issues or challenges faced during the trial.

4. Review and approve major changes to the protocol.

Trial Steering Committee will meet quarterly to review trial progress, and additional meetings can be scheduled if pressing issues arise.

Study group will also have access to WhatsApp group and Facebook closed group to exchange comments/observations and provide organizational support. Study Chief, Study Director, and Study Secretary will be available to all study participants and other collaborators 24/7. Data will be managed by each surgeon designated from collaborator centers. All designated surgeons will form data management team. Data management team will ensure the integrity and security of the trial data. The group will also play a role in assisting in the day-to-day functioning of the trial (logical support, patients management).

### Composition of the data monitoring committee, its role and reporting structure {21a}

Despite trial being a low-risk intervention, the Data Monitoring and Ethics Committee will convene biannually, or more frequently if emergent issues arise, to review trial conduct and ensure the safety and well-being of participants.

### Adverse event reporting and harms {22}

All (serious) adverse events ((S)AEs) related to study reported by surgeons or participants will be recorded. Participants in both groups will be asked in standardized manner (7–10 days, 3 months and 12 months post-op). If any major event occurs between inspections, each participant will have the opportunity to contact the Study Director or Study Secretary.

### Frequency and plans for auditing trial conduct {23}

The Project Management Group will play a vital role in the daily operations and oversight of the trial. To ensure smooth execution and address any potential challenges promptly, the Project Management Group will meet biweekly. During these meetings, trial conduct will be reviewed, potential issues will be discussed, and resolutions will be sought. The Trial Steering Group will provide strategic direction and oversight to the trial and will convene on a quarterly basis, or more frequently if required by specific circumstances. Meetings will involve reviewing trial conduct, evaluating milestones achieved, and offering guidance on upcoming phases of the trial.

### Plans for communicating important protocol amendments to relevant parties (e.g., trial participants, ethical committees) {25}

Any modifications made to the protocol after its original acceptance will be submitted to Bioethics Committee in District Health Center in Gdańsk and have to be approved by the Steering Committee and Collaborators. Modification and new information will be communicated to the included participants.

### Dissemination plans {31a}

The study investigators will inform all participants about the results of the study. The results of the study will be reported in peer-reviewed international journals and popular science articles and presented at local and international conferences. Policymakers, healthcare providers, funding bodies, and other important stakeholders, e.g., patient organizations, will be informed on the outcomes of the MEFI Trial.

### Patient Public Involvement (PPI)

The outcomes of this research should be accessible and understandable to patients as well. Project Management Group will be collaborating with patient representatives to develop a patient-friendly summary of trial’s finding. This summary will be made available on our website and will be shared with patient support groups and relevant organizations. Also, there will be a feedback mechanism wherein participants can share their experience of being part of the trial. This feedback will be periodically reviewed Project Management Group and the patient advisory group to identify areas of improvement and implement necessary changes.

## Discussion

In our opinion, flat, macroporous implants used in most laparoendoscopic inguinal hernia repairs performed in Germany (included in the Herniamed Database) are not the most appropriate for this type of procedure. While the groin operated from the anterior approach (Lichtenstein’s operation) is approximately a flat structure and thus flat implants are appropriate in this case, laparoendoscopic procedures differ in their specificity. The groin seen from behind is by no means a flat structure. Curvatures of pubic bones, iliac vessels, etc., require an appropriate implant that matches its shape. This will enable, e.g., obtaining many contact points, which in turn, by creating friction, keep the implant in its initial position. The above issues are of little importance in the case of small hernia defects. Small size of the gate (< 3 cm) does not allow the implant to move. However, in the case of large simple and complex hernias, the type of implant is a key issue, and the mere presence of such defects is a risk factor for recurrence.

In the study by Zamkowski et al., rigid spatial implants (3d Max, Dextile Anatomical Mesh, Filaprop 3d Mesh) did not change their position regardless of the increase in intra-abdominal pressure. Thus, they did not give rise to a rapid recurrence [3]. In the case of flat, macroporous implants, similar mechanical stability can only be achieved by using fixing materials, as shown by the analysis of Mayer et al. [2]. It is worth mentioning that the mesh mounting itself may involve a number of potential complications.

In addition to the obvious ones, which include the increase in the cost of the procedure and longer duration of the surgical procedure, there are many others. The literature describes the possibility of migration of the fixation with meshoma formation, adhesions, chronic postoperative pain, and infections. Each fixation itself carries a risk of damage to the surrounding tissues, including accidental nerve damage. This applies in particular to fixation with sutures, takers, or staples and has been proven in post-mortem studies [7, 8]. Finding a way to avoid the need for fixation by using dedicated implants will eliminate all of the above potential risks [1, 9–12].

Implants in the control group in our study are fixed atraumatically, using tissue glue. The weak recommendation contained in the guidelines for the treatment of hernias states that atraumatic fixation (with the use of fibrin or acrylic glue) reduces the risk of damage to the surrounding tissues [1]. There is also the issue of cost-effectiveness behind this solution—adhesives are potentially the cheapest method of fixing. Considering the above, in our study, in the control group, we adopted this method of implant attachment.

A trend is slowly emerging in the literature to use heavier implants in the case of large and complex inguinal defects treated with laparoendoscopic techniques. Some studies point to the superiority of heavier implants in the context of recurrence, especially when routine fixation is not used [13, 14]. Previously mentioned analysis of Swedish Hernia Registry showed that M3 hernia repairs are not connected with higher recurrence rate as long as larger, heavier mesh is used [4].

However, the final confirmation of the above in the conditions of a randomized clinical trial is still missing.

Confirmation in the RCT that the recurrence rate for spatial implants in M3 hernias is the same as for fixation of flat implants will be the last element necessary to apply for a change, or at least a supplement, to the international guidelines.

## Trial status

Protocol version number and date: version 2.0 date 19/01/2023. Recruitment for the trial began in February 2023 and is expected to be completed by February–March 2024.

## Data Availability

The datasets used during the current study are available from the corresponding author on reasonable request.

## Abbreviations

TAPP: Trans-abdominal preperitoneal
TEP: Totally extraperitoneal
EHS: European Hernia Society
VAS: Visual Analog Scale
ANOVA: Analysis of Variance
StPPM: Standard Pure Polypropylene Mesh
RCT: Randomized controlled trial
SAE: Serious adverse events
PPP: Preoperative progressive pneumoperitoneum
ACS: Abdominal compartment syndrome
